# Selection at a Single Locus Leads to Widespread Expansion of *Toxoplasma gondii* Lineages That Are Virulent in Mice

**DOI:** 10.1371/journal.pgen.1000404

**Published:** 2009-03-06

**Authors:** Asis Khan, Sonya Taylor, James W. Ajioka, Benjamin M. Rosenthal, L. David Sibley

**Affiliations:** 1 Department of Molecular Microbiology, Washington University School of Medicine, St. Louis, Missouri, United States of America; 2 Department of Pathology, University of Cambridge, Cambridge, United Kingdom; 3 Animal Parasitic Disease Laboratory, Animal and Natural Resources Institute, Agricultural Research Service, United States Department of Agriculture, Beltsville, Maryland, United States of America; National Institute of Genetics, Japan

## Abstract

Pathogenicity differences among laboratory isolates of the dominant clonal North American and European lineages of *Toxoplasma gondii* are largely controlled by polymorphisms and expression differences in rhoptry secretory proteins (ROPs). However, the extent to which such differences control virulence in natural isolates of *T. gondii*, including those from more diverse genetic backgrounds, is uncertain. We elucidated the evolutionary history and functional consequences of diversification in the serine/threonine kinase ROP18, a major virulence determinant in the mouse model. We characterized the extent of sequence polymorphism and the evolutionary forces acting on *ROP18* and several antigen-encoding genes within a large collection of natural isolates, comparing them to housekeeping genes and introns. Surprisingly, despite substantial genetic diversity between lineages, we identified just three principal alleles of *ROP18*, which had very ancient ancestry compared to other sampled loci. Expression and allelic differences between these three alleles of *ROP18* accounted for much of the variation in acute mouse virulence among natural isolates. While the avirulent type III allele was the most ancient, intermediate virulent (type II) and highly virulent (type I) lineages predominated and showed evidence of strong selective pressure. Out-group comparison indicated that historical loss of an upstream regulatory element increased *ROP18* expression, exposing it to newfound diversifying selection, resulting in greatly enhanced virulence in the mouse model and expansion of new lineages. Population sweeps are evident in many genomes, yet their causes and evolutionary histories are rarely known. Our results establish that up-regulation of expression and selection at *ROP18* in *T. gondii* has resulted in three distinct alleles with widely different levels of acute virulence in the mouse model. Preservation of all three alleles in the wild indicates they are likely adaptations for different niches. Our findings demonstrate that sweeping changes in population structure can result from alterations in a single gene.

## Author Summary

The determinants of virulence are rarely defined for eukaryotic parasites such as *T. gondii*, a widespread parasite of mammals that also infects humans, sometimes with serious consequences. Recent laboratory studies have established that variation in a single secreted protein, a serine/threonine kinase known as ROPO18, controls whether or not mice survive infection. Here, we establish the extent and nature of variation in *ROP18* among a collection of parasite strains from geographically diverse regions. Compared to other genes, *ROP18* showed extremely high levels of diversification and changes in expression level, which correlated with severity of infection in mice. Comparison with an out-group demonstrated that changes in the upstream region that regulates expression of *ROP18* led to an historical increase in the expression and exposed the protein to diversifying selective pressure. Surprisingly, only three atypically distinct protein variants exist despite marked genetic divergence elsewhere in the genome. These three forms of *ROP18* are likely adaptations for different niches in nature, and they confer markedly different virulence to mice. The widespread distribution of a single mouse-virulent allele among geographically and genetically disparate parasites may have consequences for transmission and disease in other hosts, including humans.

## Introduction

*Toxoplasma gondii* provides a valuable model for studying the evolution of pathogens because it is widespread and infects virtually all warm-blooded vertebrates, including companion and agricultural animals. This remarkably successful parasite is easily acquired by ingesting either oocysts (excreted by cats) or tissue cysts (in undercooked meat) [1]. These different modes of transmission allow efficient spread via water or food borne ingestion and result in very high prevalence rates of chronic infection in animals and humans [1]. Despite the presence of a sexual cycle, which occurs only in cats, the population structure can be remarkably clonal, with just three highly similar lineages predominating in North America and Europe [2]–[4]. These lineages were previously referred to as types I, II, and III (or groups 1, 2, and 3) and are referred to here as “the clonal lineages”. Estimates of the common ancestry of these clonal lineages indicates that a dramatic genetic sweep lead to the successful expansion of these lineages during the past ∼10,000 years [5]. In contrast, distinct strains from South America are more genetically variable, reflecting both asexual and sexual propagation. Phylogenetic and population genetic analyses indicate that northern and southern strains diversified during geographic separation over the past several million years [6],[7]. The three clonal lineages in the North share a monomorphic version of chromosome Ia, which appears to have arisen coincident with their origin, and which has more recently penetrated into the South [7],[8].

Comparison of interstrain polymorphisms suggests the clonal lineages originated from only a few genetic crosses between highly related parents (1–2% divergence), engendering an unusual pattern of biallelism at most loci [9]. Within these otherwise highly homogeneous genomes, a small number of polymorphic genes encode variable surface antigens (SAGs) and antigenic proteins secreted from rhoptries (ROP) or dense granules (GRA) [10]–[13]. Despite their genetic similarities, the northern clonal lineages differ markedly in their virulence to outbred laboratory mice: type I strains are acutely virulent with an estimated 100% lethal dose (LD_100_) of a single organism, while types II and III are relatively nonvirulent (LD_50_>10^5^) [14]. Mice are a natural host for *T. gondii*, and the laboratory mouse provides an excellent model for establishing differences in pathogenesis between parasite strains. Type I strain show faster rates of replications in vitro [15], enhanced migration *in vitro* and *in vivo* [16], and reach higher tissue burdens [17] in laboratory mice. While acute mortality exhibited in mice may not be directly extrapolated to other hosts, such experimental models can identify candidate factors that may influence the outcome of infection in other hosts, potentially affecting transmission and disease.

Various factors are likely to influence the outcome of human infection, including the genotype of the parasite. However, because human-to-human transmission plays little role in the evolution of *T. gondii*, any contribution of parasite genotype to pathogenesis is an indirect consequence of adaptation in other hosts. The vast majority of human cases of toxoplasmosis that have been described in North America and Europe belong to the type II genotype [4],[18]. Type II strains often cause mild infection in healthy individuals, and yet can very severe congenital infection and acute severe toxoplasmic encephalitis in AIDS patients. While relatively uncommon in the wild, type I strains have been associated with several small cohorts of human congenital toxoplasmosis and opportunistic infection in AIDS patients [19],[20], suggesting type I strains may be more likely to cause disease in permissive hosts. In other regions of the world, genetically more diverse strains have been associated with severe infection. For example, atypical genotypes of *T. gondii* predominate in French Guyana where severe infections have been described in otherwise healthy adults [21],[22]. Divergent genotypes of *T. gondii* have also been associated with severe ocular toxoplasmosis in southern Brazil [23] and in the United States [24]. Notably, strains from both of these South American regions are acutely virulent in the mouse model, similar to the type I lineage.

The marked phenotypic differences expressed in the murine model have been utilized to explore the contribution of parasite genotype to pathogenesis. Excellent forward and reverse genetic systems have recently supported genome-wide analyses of the factors that control phenotypic differences between the clonal lineages. Independent screens have converged on secretory proteins, discharged from apical organelles called rhoptries, as key modulators of both host gene transcription [25] and of acute virulence in the mouse model [26],[27]. During host cell invasion, the parasite discharges the contents of rhoptries into the host cell, thereby delivering a variety of kinases, phosphatases, and other potential effectors directly into the host cell [28],[29]. Genetic mapping studies have shown that the primary determinant of acute mouse virulence in type I strains of *T. gondii* is a polymorphic rhoptry kinase known as ROP18, which relies on serine/threonine (S/T) kinase activity to promote growth [30], and enhance virulence in the mouse model [27]. *ROP18* and other polymorphic ROPs have been implicated in the more subtle differences in pathogenicity that occur between types II and III strains in the mouse model [25],[26]. Despite these advances, the phenotypic consequences of variation in *ROP18* in genetically diverse, natural isolates of *T. gondii* remain uncertain. Here, we examined the contribution of genetic variation at ROP18 to the natural variation in mouse virulence and the population structure of *T. gondii*.

## Results/Discussion

### *ROP18* Is among the Most Divergent Genes in *T. gondii*

**Figure 1:**
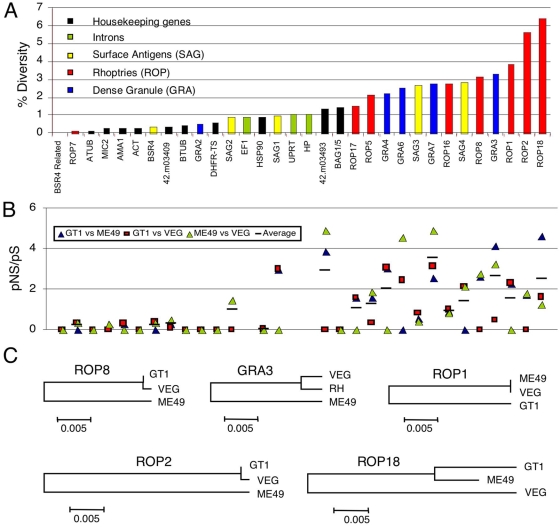
Diversity among *T. gondii* genes varies by category of coding function. A) Sequence diversity of genes and intronic sequences between representatives of type I (GT1), type II (ME49) and type III (VEG) strains of *T. gondii*. Loci listed at the bottom in rank order, annotations in Table S1. B) Ratio of pNS/pS as an indication of positive selection. Pairwise comparisons of sequences between strains as indicated. C) Neighbor joining trees reconstructed from variation in the five most divergent genes. Scale = substitutions per site.

The clonal lineages of *T. gondii* are 98% identical at most loci, reflecting the close similarity of parental lineages that gave rise to them [9]. Within lineage polymorphism is significantly lower, on the order of 1 change in 10,000 base pairs (bp), consistent with the very recent ancestry of the three clonal lineages [5]. In comparing inter-lineage diversity, certain genes exhibit more polymorphism and hence might underlie phenotypic variation between strain types. We compared the diversity of genes encoding polymorphic surface and secretory antigens (i.e. *SAGs*, *ROPs*, *GRA*s) with *ROP18* and other putative virulence factors identified by previous genetic mapping studies [25]–[27]. For comparison, we included several housekeeping genes and introns, previously used for phylogenetic studies [7]. Polymorphism was characterized in a total of 32 separate loci using the recently completed genome sequences from archetypal strains of the type I, II and III (Table S1). Four of the five most variable genes identified belonged to the ROP family, with *ROP18* being the most diverse (Figure 1A). Interestingly, three of these belong to the ROP2 family of proteins that contain a serine threonine kinase domain [31]. While most are predicted to be catalytically inactive, ROP18 retains activity [30] and this is required for its virulence enhancing potential [27]. Although variable, most of the *ROP*s showed little evidence of positive selection (based on the proportion of nonsynonymous (pNS)/synonymous (pS) substitutions) regardless of whether they are predicted to be pseudokinases (i.e. *ROP2*, *ROP5*) or active members of this family (i.e. *ROP16*, *ROP17*). In contrast, strong evidence of positive selection was identified for *ROP18* and several polymorphic dense granule (GRA) antigens (i.e. *GRA3*, *GRA6*, *GRA7*) [10],[12],[13] (Figure 1B). While most genes conformed to the expected biallelic pattern of inheritance [9], three atypically divergent alleles were found at *ROP18* (Figure 1C). *ROP18* is thus distinguished by uncommonly abundant polymorphism, which may be at least partially attributed to strong, positive selection.

### Only Three Common *ROP18* Alleles Account for Its Exceptional Genetic Diversity

**Figure 2:**
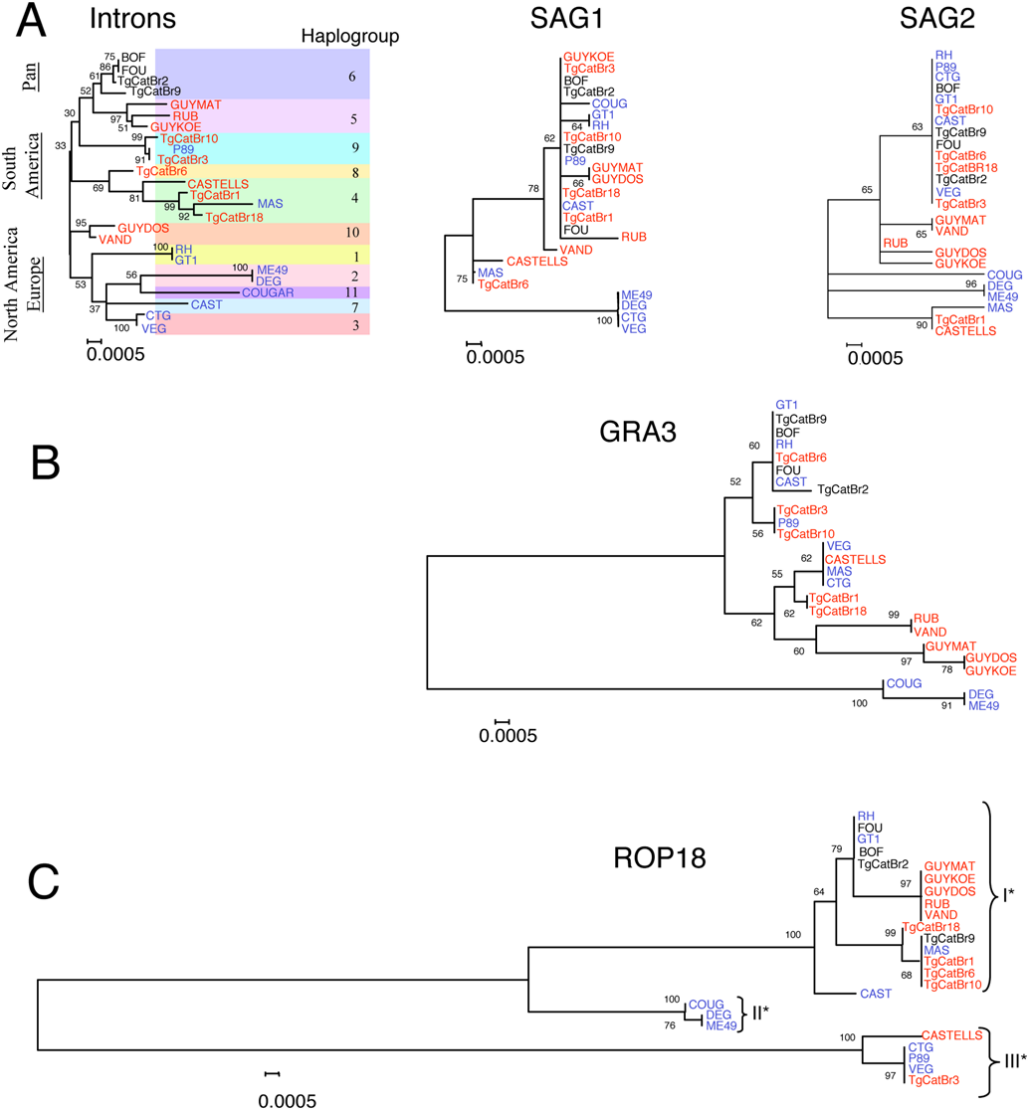
Phylogenetic analysis reveals *ROP18* is highly divergent relative to other loci. A) Combined analysis of 8 introns from 25 strains defines 11 major haplogroups of *T. gondii* that show strong geographic segregation. Separate gene trees for the antigens *SAG1* and *SAG2* show overall lower diversity of alleles but approximate the same level of divergence seen in the combined intron tree. B) The highly polymorphic gene *GRA3* shows deeper branching of multiple major alleles, which are not associated with geographic region or virulence. C) In contrast the much higher genetic divergence of *ROP18* is partitioned into only three major alleles (denoted by I*, II*, and III*). Neighbor-joining analysis with 1000 bootstraps (values shown at major nodes). Color-coded by continent of origin: North America-Europe (blue), South America (red), widespread (denoted as Pan, black).

Previous studies have shown that *ROP18* plays a prominent role in mediating virulence differences between laboratory isolates of clonal lineages of *T. gondii* [26],[27], yet its role in the pathogenesis of natural isolates remains unknown. To compare the genetic diversity of *ROP18* in *T. gondii*, we characterized 25 representative isolates representing the 11 previously defined haplogroups, sampled from animals and humans in North America, Europe, and South America (Table S2). Genetic diversity in *ROP18* was compared within eight introns from five unlinked loci, two surface antigen genes (i.e. *SAG1* and *SAG2*), the secreted protein *GRA3*, and two house keeping genes (i.e. *actin and β-tubulin*). Phylogenetic analysis of the intron sequences revealed 11 main haplogroups, which occupy particular geographic ranges (Figure 2A), consistent with previous findings [7]. Although individual gene tress reconstructed from the less variable *SAG1* and *SAG2* sequences resolved fewer lineages, these were generally concordant with the topology established from intron sequences (Figure 2A). *GRA3*, which was one of the most highly polymorphic genes identified in Figure 1, showed greater divergence than the *SAG*s (Figure 2B). The several identified alleles of *GRA3* did not correlate with either geographic distribution or virulence in murine infections (Figure 2B).

The extent and distribution of variation evident in *ROP18* alleles contrasts sharply with the patterns seen in other genes. Firstly, only three major lineages of *ROP18* are evident, each typifying one of the three clonal lineages that predominate in North America and Europe (designated as ROP18I*, ROP18II*, and ROP18III* alleles) (Figure 2C). Secondly, diversity in *ROP18* is an order of magnitude greater than that of *SAG1* and *SAG2*, which themselves may experience diversifying selection [11]. Finally, the vast majority of South American isolates share the type I* allele characteristic of the highly virulent North American lineage. In total, members of 8 different haplogroups were found to express ROP18I* alleles. Collectively, these findings reveal that the history of *ROP18* is very different from that of the rest of the genome, resulting in the survival of only three, highly divergent allelic types.

### Polymorphism in *ROP18* Is Asymmetrically Distributed

**Figure 3:**
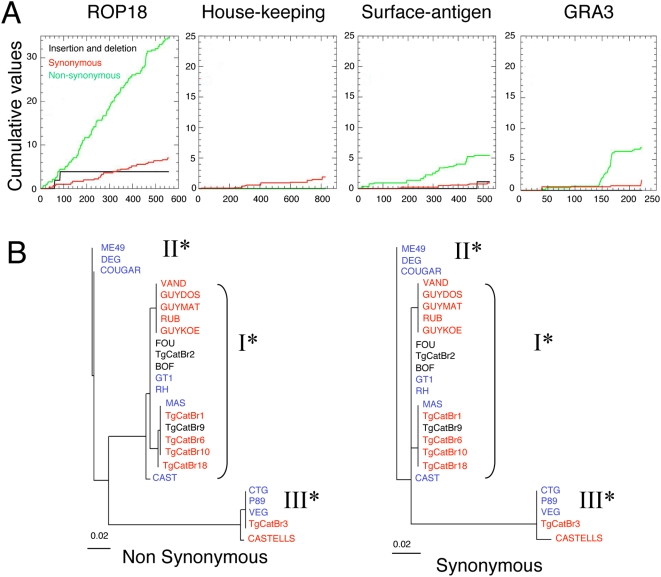
Polymorphism of *ROP18* between major alleles. A) Cumulative nonsynonymous (NS) and synonymous (S) polymorphisms in *ROP18* are significantly higher than surface antigens, *GRA3*, or housekeeping genes. B) Phylogenetic analysis of *ROP18* analyzed separately for nonsynonymous changes (left) and synonymous changes (right) reveals a strongly asymmetric pattern. The major difference between types I* and II* is due to nonsynonymous changes. Scales = substitutions/site.

Divergence between the clonal lineages extends for approximately 30 kilobase pairs (kb) surrounding *ROP18*, the peak divergence occurring in the coding sequence (Figure S1). To better understand whether alleles of *ROP18* have experienced atypical evolutionary pressures, we classified the extent of synonymous (S) vs. nonsynonymous (NS) mutations at this and several other genes, including housekeeping genes (i.e. *actin* and *β-tubulin*), the surface antigens (i.e. *SAG1*, *SAG2*), and *GRA3*. This comparison was based not only on the clonal lineages, but on all strains that were grouped by their ROP18 alleles into major types as defined in Figure 2B. Both S and NS substitutions were much more frequent in *ROP18* than in surface antigens (*SAG*s), *GRA3* or housekeeping genes (Figure 3A). Such mutations were distributed throughout the *ROP18* gene and were not due to changes in the usage of nucleotides or codons (data not shown). The ratio of pNS/pS substitutions, which indirectly measures the selective advantage of mutations incurring amino acid substitutions, was greater in *ROP18* (∼2.5 average pairwise comparison for I* vs. II*, I* vs. III*, and II* vs. III*)) than in *SAG1* (∼1.29 average pairwise comparison). This was especially evident when comparing ROP18I* and ROP18II* (3.54) while a higher proportion of S changes in ROP18III* resulted in lower ratios (average value of pNS/pS = 1.32 for ROP18III* vs. ROP18I* and ROP18III* vs. ROP18II*, (Table S3)). Phylogenic trees drawn based on S *vs.* NS differences illustrate the marked deficiency of synonymous changes differentiating ROP18I* and ROP18II* from each other (Figure 3B). These data imply markedly different selective pressures have shaped the divergence among these three *ROP18* alleles over time.

Deep divergence also characterizes other members of the ROP2 family, which contains a number of predicted pseudokinases of unknown function [31]. Although the ancestry described here for ROP18 may reflect that of the ROP2 family more generally, ROP18 is notable among this group for showing evidence of strong diversifying selection. ROP18 is also the only member of this group known to be catalytically active and directly implicated in virulence, hence it is reasonable to assume that selection on this locus had important functional consequences.

### ROP18I* Alleles Confer Acute Virulence in the Murine Model

**Figure 4:**
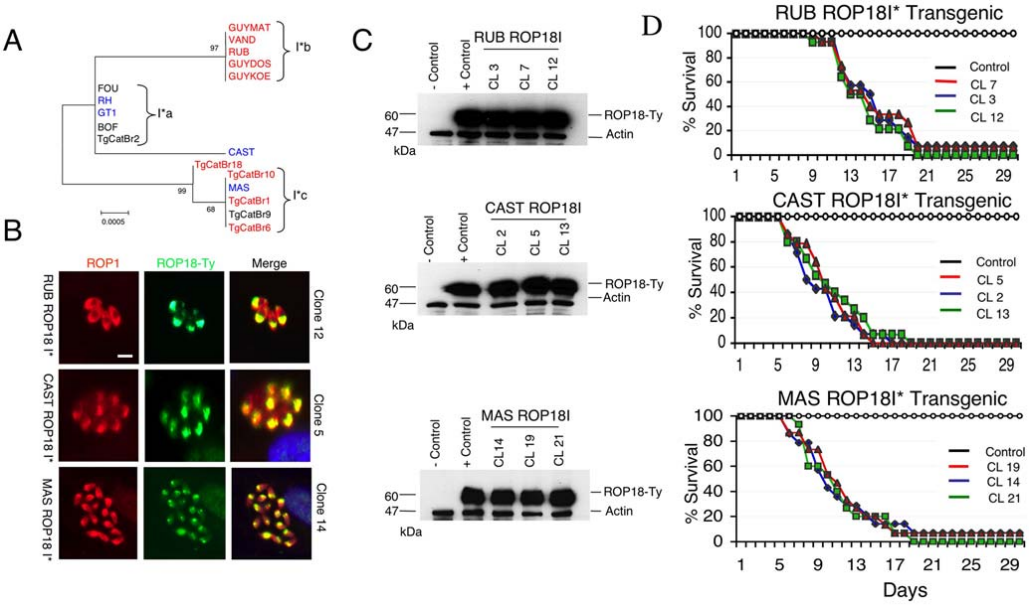
Newly discovered ROP18I* alleles confer acute virulence. A) Phylogenic analysis of the ROP18I* alleles reveals three major groups (denoted I*a, I*b, and I*c) plus a single node for strain CAST. Tree depicts the same data as in Figure 1B, plotted with an expanded scale. B) Expression of RPO18I* alleles in transgenic lines and colocalization with ROP1 in the rhoptries was confirmed by immunofluorescence staining. ROP18I* alleles were tagged with Ty (green) and visualized using mAb BB2. Endogenous control detected with rabbit anti-ROP1 (red). Representative clones are shown for each allele subtype of ROP18I*. Scale bar = 5 microns. C) Three separate transgenic lines from representative strains as shown, expressed ROP18I* alleles at comparable levels to the previously described ROP18Ia V1 clone (+control) [27]; vector only control (−control). ROP18 was detected with mAb to the Ty-tag, rabbit anti-actin provided as loading control. D) Mouse survival assays demonstrating that expression of ROP18I* alleles in the type III background resulted in increased mortality. Cumulative mortality following challenge with 10, 100, and 1,000 parasites for each of three clones is plotted as a single line per clone (CL). Control is transgenic type III strain containing only the Ble vector.

Previous studies have shown that *ROP18* is under expressed in the type III lineage, providing a suitable background for testing gain-of-function by transgenic expression [27]. Expression of the type I allele of *ROP18* in the type III background reconstitutes the acutely virulent phenotype characteristic of the type I clonal lineage [27]. While additional ROP18I* alleles share similarities with the virulent allele from the clonal type I tested previously (corresponding to group I*a) [27], they also differ significantly from one another, , presumably due to genetic drift (Figure 4A). Hence, we undertook a similar reverse genetic analysis with the newly identified subtypes of ROP18I* in order to determine if they were also capable of conferring acute virulence or if they had lost this trait due to mutation. We chose three representative isolates for this analysis from different branches of the ROP18I* tree that have not been previously analyzed; including RUB (ROP18I*b), MAS (ROP18I*c) and CAST. These strains are all acutely virulent in mice (Table S2). We established three separate transgenic lines from each of the parasite isolates, using standard methods for epitope tagging and isolation of stable lines, as described previously [27]. Expression and localization to the rhoptries was confirmed by immunofluoresence staining of the Ty-epitope tag in reference to the endogenous rhoptry protein ROP1 (Figure 4B). Expression levels comparable to the previously derived transgenic line expressing ROP18Ia* (V1 clone, described previously [27]) were confirmed by western blotting (Figure 4C). Challenging adult outbred mice with these transgenic parasites confirmed that the new alleles of ROP18I* conferred acute virulence. Mice died within 15–20 days, even when inoculated with few parasites (i.e. 10 tachyzoites) (Figure 4D). Collectively, these data indicate that the majority of haplogroups of *T. gondii* express ROP18I* alleles, and that these different variants all confer acute virulence in the mouse model.

This finding is significant because it indicates that amino acid substitutions conferring increased virulence have become fixed in a lineage of ROP18I*, which is expressed by a wide range of isolates. The widespread distribution of ROP18I* alleles indicates that this gene and the traits it controls, confers a selective advantages in certain settings.

### Differences in the Expression of *ROP18* Largely Explain Differences in Murine Virulence

**Figure 5:**
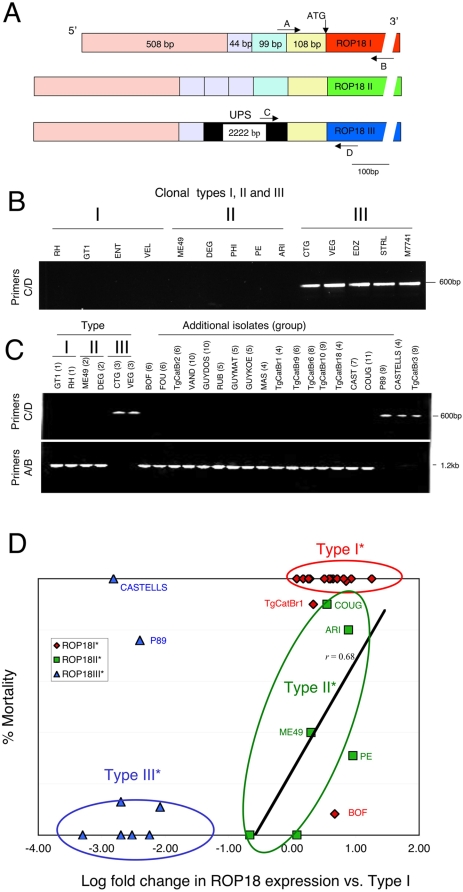
Expression levels of *ROP18* correlate with acute virulence. A) Schematic of *ROP18* coding and upstream regions based on sequence analysis of the three clonal lineages. A ∼2 kb upstream segment (UPS) shown in black (not to scale) is unique to the type III genotype. Type I and II are similar, although Type II contains three tandem repeats of a 44 bp region that is only found once in type I. B) PCR analysis from genomic DNA of clonal isolates (group) demonstrating only type III strains (group 3) contain the UPS region (primers C/D). C) Differential amplification of the upstream regions from a variety of strains reveals that most strains lack the UPS (positive with primers A/B) and resemble types I and II, while the UPS is limited to types 3, 9 and CASTELLS. D) Acute mortality in outbred mice (% cumulative mortality) is correlated with allele-type and level of expression of *ROP18* as determined by qPCR. Strains include those in Table S2 where mortality data is available. Correlation of expression level in type II* stains is plotted as a linear regression, r = 0.68.

Previous genetic crosses between laboratory strains of clonal types I and III, attributed most of the variance in virulence to expression differences in *ROP18* [26],[27]. This difference in expression has been ascribed to an additional DNA segment upstream of the *ROP18* gene in the type III lineage that alters transcription [32]. To examine the contribution of *ROP18* expression to the variation in virulence among natural isolates, we examined the surrounding genomic regions and tested expression by real-time quantitative PCR (qPCR). Substantial differences in the upstream regions of *ROP18* were evident when the three clonal lineages were compared (Figure 5A). Direct DNA sequencing revealed that Types I and II were highly similar, aside from the tandem duplication of three copies of a 44 bp block in type II, while this region only occurs once in type I (Figure 5A). In contrast, the type III lineage contains a ∼2 kb segment of DNA upstream of the *ROP18* gene that was not present in types I or II (Figure 5A). There is no similarity of this upstream segment (UPS) to other regions of the genome nor is there any homologous sequence in NCBI (data not shown). Using specific PCR primers that distinguished the upstream region of types I and II (primers A/B) from type III (primers C/D), we demonstrated that they amplified mutually exclusive fragments from genomic DNAs derived from a set of representative clonal isolates (Figure 5B,C). This analysis confirmed that the UPS is unique to the type III lineage (Figure 5B). Further analysis of a wider collection of strains from other haplogroups identified the UPS only in members of type III (group 3), group 9, and a single member of group 4 (i.e. CASTELLS). All other strains lacked the UPS (Figure 5C). Sequencing the coding regions of ROP18 III* alleles confirmed that these were always flanked by the UPS, which was invariably lacking in ROP18 I* and ROP18II* alleles (data not shown).

We also tested all of the above strains for acute virulence in the mouse model described previously [27], and evaluated expression based on qPCR. Strains containing the UPS expressed low levels of *ROP18* and were nonvirulent in outbred mice, whereas the opposite was true of parasites lacking the UPS (Figure 5D). In total, 17/18 type I* strains were acutely virulent while 6 of 8 type III* were avirulent (Table S2). Rare exceptions to this pattern (i.e. BOF for type I*; P89 and CASTELLS for type III*) were not due to differences in either the coding or the upstream regions of *ROP18* (sequencing data not shown), and presumably arise from other genetic differences. For example, BOF contains a type I* allele, lacks the UPS, and shows high expression of *ROP18*, yet is avirulent in the mouse model, presumably due to a defect in some other pathway needed for efficient infection. Importantly, this strain was isolated from an AIDS patient, suggesting it may only be pathogenic in immunocompromised hosts (Table S2). Conversely, P89 and CASTELLS contain a type III allele and the corresponding UPS, consistent with their low expression of *ROP18*, yet they both show high levels of virulence. Presumably this is the result of another favorable combination of genes that enhance pathogenicity, as these strains are not conventional members of the type III clonal lineage, but rather divergent isolates. Importantly, the variation in expression level among type I or type III strains differed by more than an order of magnitude, without altering their respective phenotypes (Figure 5D). Whereas virulence in ROP18I* and ROP18III* expressing strains tended to be all or none, virulence among ROP18II* expressing strains increased with the expression level (Figure 5D). Among strains expressing type II* alleles, expression level and mortality were highly correlated (r = 0.68) as shown by linear regression analysis (line in Figure 5D). Consistent with this, previous reports indicate that over-expression of the type II allele in the type III background leads to a dramatic increase in virulence [26]. The low level of expression of ROP18III* may be finely tuned to the genotype as a whole, since it has thus far not been possible to over-express the type III allele or to disrupt *ROP18* by homologous recombination (data not shown). Overall, the combination of allele and expression level of *ROP18* correlates with acute virulence in 30 of 33 strains included here. Hence, differences in acute virulence among these isolates can be largely explained by the allele of *ROP18* and the extent of its expression.

### DNA Rearrangements Were Associated with Upregulation of *ROP18* Expression

**Figure 6:**
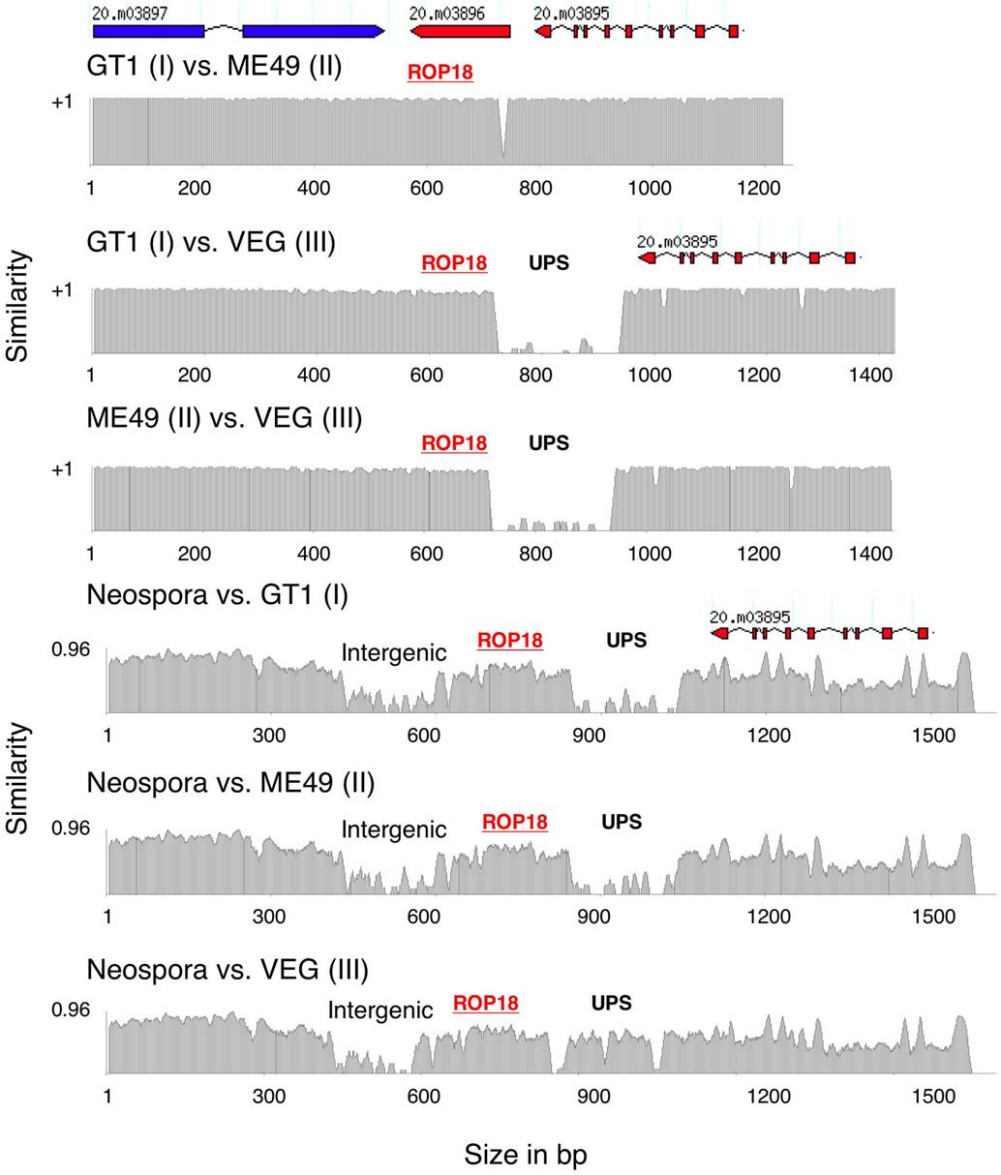
Evolutionary history of the upstream region in *T. gondii* compared to the out-group *N. caninum*. Pairwise sequence comparisons of genomic sequences between *T. gondii* strains and *N. caninum* showed the region surrounding ROP18 is highly syntenic with preservation of gene order and intergenic regions in *N. caninum*. The upstream region (UPS) of type III ROP18 (VEG) is found in *N. caninum* but is absent in type I (GT1) and type II (ME49), indicating that it was retained by type III* but lost in the ancestor to types I* and II*. The small region of dissimilarity between type I (GT1) and type II (ME49) corresponds to a slight difference in the rearrangements that presumably occurred in these lineages as shown in Figure 5A. X-axis is length in bp. Y-axis indicates the similarity values between two strains.

The striking increase in *ROP18* expression in the absence of the UPS, previously established for laboratory strains [32], also characterizes our broader sample of natural isolates. Two scenarios could explain how such difference came to be: 1) the UPS was inserted in the ancestor that gave rise to group 3 (type III) and group 9, or 2) the UPS was deleted from (or rearranged in) the common ancestor to the ROP18I* and ROP18II* alleles. To test these two models, we used an out-group comparison to infer the ancestral condition of this genomic region, employing the homologous sequence from the animal pathogen *Neospora caninum* [33]. Previous estimates based on small subunit RNA and the internal transcribed spacer (ITS) region of the ribosomal RNA indicate *T. gondii* shared a common ancestor with *N. caninum* ∼10 million years ago [5]. A syntenic block encompassing *ROP18*, and the two genes flanking it, was identified in *N. caninum* (available whole genome sequence assembly generated by The Wellcome Trust Sanger Institute (http://www.sanger.ac.uk/sequencing/Neospora/caninum/), allowing comparison to *T. gondii* (Figure 6). Like ROP18III*, the *ROP18* gene in *N. caninum* included a UPS region not present in either ROP18I* or ROP18II* (Figure 6). Clustal analyses revealed that *N. caninum* was ∼54% identical to ROP18III* in this upstream region, and ∼68% identical in the coding region. Thus, the UPS region, evidently present in the common ancestor of all *T. gondii*, was lost by deletion or rearrangement in the ancestor to the more recently derived ROP18I* and ROP18II* alleles.

### *ROP18* Shows a Biphasic Ancestry Associated with Expansion of Virulence

The high degree of divergence among allelic lineages of *ROP18* suggests it has either undergone faster change or evolved over a longer period of time, relative to most regions of the genome. To differentiate among these alternative explanations, we compared the extent intra-lineage variation in *ROP18* to that present in several surface antigen genes in 17 representative members of the clonal lineages. *ROP18* did not contain more polymorphism than did other genes or introns, suggesting no elevation in its rate of molecular evolution (Table S4). Indeed, as previously derived from putatively neutral loci such as introns [5], the scarcity of unique polymorphisms in *ROP18* within each of the three clonal types is consistent with the model that they originated within the last ∼10,000 years (Table S4). Because *ROP18* has not been changing at an especially rapid rate, our data instead suggest that the principal alleles have been maintained for an especially long period of time.

**Figure 7:**
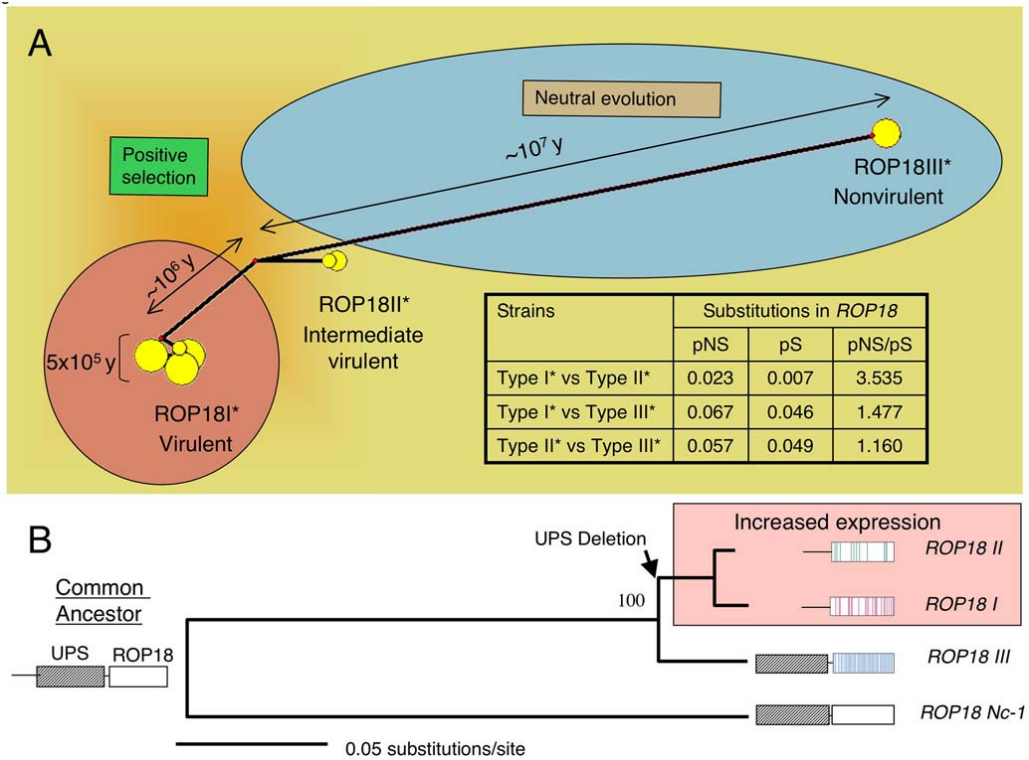
Evolutionary history of *ROP18*: changes in expression levels of *ROP18* correlate with acute virulence. A) Network analysis of *ROP18* reveals two different periods of evolution. A long period of nearly neutral evolution differentiated type III* from the ancestor of types I* and II*. More recently, the types I* and II* diverged under a period of strong selection. Acute virulence is associated with the successful expansion of the type I* alleles. Table includes proportion of nonsynonymous (pNS)/synonymous (pS) substitutions for *ROP18* (see also Table S3 for additional ratios). Age estimates are based on synonymous changes in ROP18 from the data in Table S5. B) Out-group rooting using the *ROP18* homologue in *Neospora caninum* confirms that the type III* lineage has undergone a longer period of independent evolution than have types I* and II*, which shared a more recent common ancestor. Neighbor joining analysis based on the coding regions of *ROP18*. Bootstrap values based on 1000 replicates. Color bars in the *ROP18* models for each strain type denote the positions of allele-specific SNPs.

Network analysis of the *ROP18* genes from the complete set of strains confirmed that the sequences cluster as three discrete nodes (Figure 7A). To estimate when the major *ROP18* alleles began diverging, we employed a model based on the accumulation of synonymous mutations and estimated the time since a common ancestry based on several assumptions for an average neutral mutation rate. These analyses revealed that the ROP18 III* allele shared a common ancestor ∼10 million years ago, while ROP18I* and ROP18II* diverged ∼1 million years ago (Figure 7A, Table S5). Although these age estimates are based on synonymous sites that are not expected to be under direct selection, it is still possible that diversifying selection acting on nearby nonsynonymous sites could have inflated the coalescence estimate. Despite uncertainty about the absolute ages of these alleles, two periods diversification are nonetheless evident. Over a relatively long initial period, primarily neutral substitutions accumulated between ROP18 III* and the common ancestor of ROP18I* and ROP18II*. A later period of diversifying selection drove expansion of ROP18I* and ROP18II* to differ markedly in their amino acid composition (pNS/pS ratio = 4.66) (Figure 7A, Table S3). The majority of extant haplogroups (8 of 11 studied here) are typified by ROP18I*, demonstrating that this allele has successfully expanded since its relatively recent origin.

### A Model for Evolution of Virulence by Alterations at a Single Locus

Our data are consistent with a model that the common ancestor of the more closely related ROP18I* and ROP18II* alleles lost the expression-suppressing UPS that occurs in ROP18III* and in the out-group homologue, *ROP18Nc-1* (Figure 7B). The resulting DNA deletion or rearrangement lead to upregulation of *ROP18* expression in ROP18I* and ROP18II* alleles. Subsequent expansion of these alleles substantially shaped the population structure of *T. gondii*. The highly virulent ROP18I* allele predominates among known haplogroups, especially those in South America, whereas the type II* allele (conferring intermediate virulence) typifies most human and animal infections in North America and Europe. Each of the three highly divergent alleles of ROP18, conferring markedly different virulence levels in laboratory mice, have been maintained over long periods of time, suggesting that they confer selective advantages in particular environmental circumstances.

Strains harboring ROP18I* have clearly enjoyed recent evolutionary success, as they characterize 8 of 11 haplogroups studied here, including a number of recently derived subgroups that appear to be expanding. However, it is doubtful that the acute virulence as expressed in the laboratory mouse model would be adaptive in natural hosts. Laboratory mice, while a convenient model, are likely more susceptible than many species that are natural hosts of *T. gondii*. For example, other rodents (*Peromyscus* spp., *Rattus* spp.) survive infections with type I strains of *T. gondii* and develop chronic infection [34],[35], promoting eventual transmission to other hosts. Thus, a reasonable hypothesis is that while ROP18I* leads to mortality in especially vulnerable hosts, such as the laboratory mouse, it instead facilitates the establishment of infection in resistant hosts, thus enhancing transmission. By contrast, type II strains are intermediate in virulence in laboratory mice, express a proclivity for developing into tissue cysts [36], and modulate host immune responses [25],[37],[38]. Collectively, such adaptations may promote the establishment of chronic infection (although this genotype has also been associated with immunopathology in several models [39],[40]). Clearly enhanced pathogenicity is not always advantageous to parasite fitness. Adaptation to an alternative niche may explain the long-term persistence of lineages expressing ROP18III*. While the ROP18III* allele is expressed at very low levels, it is not a pseudogene and despite its very high divergence, it preserves all of the essential residues in the S/T kinase domain necessary for activity (data not shown). This suggests that low levels of expression may be an adaptation for infection in some particular hosts, potentially those that are highly susceptible to infection. Defining the precise advantages of each of these alleles will require further studies of the transmission between natural hosts and a better understanding of the population genetic structure in the wild.

Mathematical modeling studies predict that pathogens may evolve greater virulence provided that doing so does not reduce their potential for transmission [41]. A clear relationship between enhanced growth, transmission, and virulence has previously been demonstrated in other pathogens, including malaria [42]. Typically the underlying molecular mechanisms for such adaptations are not known. Our findings indicate that changes in the expression of a single gene, that has undergone strong diversifying selection, has markedly increased virulence as monitored in laboratory mice. Understating pathogenic determinants of *T. gondii* in model systems, will facilitate future studies to more precisely evaluate the contribution of parasite genotype and specific genes, to transmission in natural hosts and potentially for human disease.

## Materials and Methods

### Comparison of *T. gondii* Gene Diversity

Sequences of 32 loci from representative genomes of clonal lineages type I, II and III, were downloaded from the *T. gondii* genome database (www.toxodb.org). These 32 loci include surface antigens, secretory proteins such as rhoptries, and dense granules, as well as housekeeping genes and introns that are presumed not to be undergoing positive (diversifying) selection (Gene ids given in Table S1). Clustal X/W [43] was used to align the sequences to calculate the percentage diversity, expressed as changes per 100 bp. The proportion of synonymous and nonsynonymous changes in coding regions were calculated using the Synonymous Nonsynonymous Analysis Program (SNAP) [44]. Neighbor-Joining phylogenetic trees were constructed using Molecular Evolutionary Genetic Analysis (MEGA) Version 3.1 [45].

### Sequencing and Phylogenetic Analysis of *T. gondii* Strains

Twenty five strains representing the 11 previously described haplogroups of *T. gondii* found in North America, Europe and South America were chosen for analysis [7]. These isolates were selected from more than 300 that have been previously genotyped based on the following criteria: known source (host and geography), passage history, mutlilocus genotyping and assignment to haplogroups, phenotypic data in animals. Parasites were propagated as tachyzoites in monolayers of human foreskin fibroblasts (HFF) [5] and lysates were made for PCR-based sequencing [7]. The frequency of single nucleotide polymorphisms (SNPs) were determined by sequencing three classes of loci: 1) noncoding regions consisting of 8 introns from 5 unlinked loci, 2) the housekeeping genes *β-TUB* and *TgActin* and 3) the surface antigen genes *SAG1* and *SAG2* in addition to coding regions of *ROP18* (Gene ids given in Table S1). Sequencing was conducted on PCR amplified templates from each strain using BigDye cycle sequencing (Applied Biosystems, Foster City, CA). Sequences were aligned with Clustal X/W [43] and phylogenetic trees were derived using Neighbor-Joining in Molecular Evolutionary Genetics Analysis (MEGA), Version 3.1 [45]. P-distances were calculated for sequence pairs after removal of insertions and deletions in 1000 bootstrap replicates. Consensus trees were drawn with an arbitrary root and a scale length of 0.0005 substitutions/site.

### Cumulative Values of Synonymous and Nonsynonymous Changes

Coding sequences were analyzed for cumulative values of polymorphisms using the Synonymous Nonsynonymous Analysis Program (SNAP) [44], based on the method of Nei and Gojobori 1986 [46] for conducting all pairwise comparisons of sequences in an alignment, and incorporating a statistic developed by Ota and Nei 1994 [47]. Ambiguous codons and insertions were excluded. Phylograms of ROP18 based only on either synonymous or nonsynonymous substitutions were performed using maximum likelihood analysis in HyPhy [48] to infer rates of heterogeneity and positive selection. These analyses were conducted assuming the codon frequency matrix MG94xHKY85_3x4 and using default setting for other values.

### Cloning of ROP18 Alleles

The *ROP18* gene was PCR amplified from genomic DNA of the *T. gondii* strains RUB [21], CAST (ATCC# 50868) and MAS (ATCC# 50870) and epitpoe tagged at the C-terminus using the Ty-epitope tag, detected by mAb BB2 [49]. Plasmids expressing C-terminal Ty-tagged ROP18 alleles were expressed under the control of the TUB promoter in the pTUB-Ty vector described previously [27]. The CTG strain (type III) was electroporated with 100 µg of pROP18I* plasmid DNAs and 10 µg of pSAG1/ble/SAG1 in cytomix buffer, as described previously [50]. Clones were isolated by single cell cloning in 96-well plates containing HFF cells following two rounds of selection with phleomycin [50]. Individual clones were screened for expression of the Ty tag by immunofluorescence and western blotting. A parallel transfection containing only the backbone plasmid was used to isolate a control transformant (denoted as CTG-Ble).

### Expression Analyses of ROP18 Alleles

ROP18 was localized in transgenic parasites by immunofluoresence labeling using the mAb BB2 to the Ty tag [49] followed by secondary antibodies conjugated to Alexa 488 and counter staining with rabbit anti-ROP1 followed by secondary antibodies conjugated to Alexa 594, as described previously [27]. Slides were washed in PBS and mounted in Vectashield with 4′,6-diamidino-2-phenylindole (DAPI) (Vector Laboratories, Inc, Burlingame, CA), examined with a Zeiss Axioscope (Carl Zeiss Inc, Thornwood, NY), and images were acquired with an AxioCam CCD (Zeiss) camera using Axiovision software v4.0 (Zeiss) and processed using Photoshop v7.0.

Proteins were resolved on 10% polyacrylamide gels using Laemmeli running buffer, transferred onto a nitrocellulose membrane by semi-dry electrotransfer and blocked in PBS containing 5% nonfat dry milk, 5% goat serum and 0.05% Tween 20. Blots were incubated for 1 hr at room temperature with mAb BB2 anti-Ty antibody (1∶1000) and M03792 rabbit antibody against actin (1∶2000) as a loading control. After washing, the membrane was incubated for 1 hr at room temperature with goat anti-mouse IgG and goat anti-rabbit IgG conjugated to horseradish peroxidase (HRP) (1∶10,000) (Jackson ImmunoResearch Laboratories, Inc., West Grove, PA). The membrane was washed for 30 min and the antibody complexes were revealed by chemiluminescence using enhanced chemiluminescence (ECL SuperSignal; Pierce Biotechnology, Rockford, IL).

### Mouse Virulence Assays

Animals were maintained in an AAALAC-approved facility and experiments were done under approval from the Washington University Animal Care Committee.

Acute virulence was monitored by determining cumulative mortality after i.p. injections of 10, 100, or 1000 tachyzoites into groups of 5 outbred CD1 mice/dose, as defined previously [27]. Animals were monitored for 30 days and any surviving animals were serologically tested by Western blot against whole tachyzoite lysate from the RH strain at 1∶1000 dilution of serum and 1∶10,000 dilution of goat anti-mouse IgG HRP (Jackson ImmunoResearch Laboratories, Inc., West Grove, PA). Signals were detected by ECL using SuperSignal (Pierce Biotechnology, Rockford, IL). Samples were compared to uninfected and chronically infected mice. Cumulative mortality was defined as the number of deaths/number of animals infected (those that died or were seropositive) and plotted as a single line for all doses combined for each transgenic clone.

### PCR-Based Analysis of the Upstream Region of ROP18

DNA lysates were prepared from freshly egressed parasites, as described previously [7]. To identify the presence of the insert upstream of *ROP18* in *T. gondii* strains, the target DNA sequence was amplified by PCR in a PTC-100 Thermal Cycler, (M.J Research, Reno, NA) using primer pairs specific for detecting the presence (Insert ROP18-F 5′ - CACAGCATGAGC TTAAGAGTTG -3′ (Primer C) and Insert ROP18-R 5′ - CACCGCAAGACAGGCTGTCTTC - 3′ (Primer D)) or absence (ROP18-F 5′ - CTAGCCACGCTATGCACCTCT - 3′ (Primer A) and ROP18-R 5′ - GCAAGTCACGCATAGTCTCATC -3′ (Primer B)) of the insert. Each reaction was carried out in 25 µl of volume containing 10× PCR buffer, 25 mM MgCl_2_, 2.5 mM dNTPs, 50 µM each of the forward and reverse primers, 5 U/µl of DNA Taq polymerase (Sigma) and 2 µl of DNA lysate of each of the strains. The reaction mixture was first heated to 95°C for 5 min, followed by 35 cycles of 94°C for 30 sec, 56°C for 30 sec and 72°C for 2 min. Upon completion, 5 µl of the PCR products were examined by electrophoresis in 2% agarose gel containing 0.3 µg/ml ethidium bromide and visualized under UV light.

### qPCR Analysis of ROP18 Expression

Total RNAs were isolated by TRIzol treatment (Invitrogen, Carlsbad, CA) of freshly harvested parasites and RNA concentrations determined by absorbance at 260 nm. RNAs (3 µg/µl) were transcribed into cDNA using 50 µM oligo (dT)_20_ and 200 units of SuperScipt III reverse transcriptase (RT) (Invitrogen, Carlsbad, CA) in a volume of 20 µl following the manufacturer's protocol. For negative controls, water was added instead of RT. Real-time quantitative PCR (qPCR) was performed using a SmartCycler (Cepheid, Sunnyvale, CA) in a 25 µl reaction volume containing SYBR green Supermix (Clontech, Mountain View, CA). *ROP18* was detected using the primers ROP18-579-F (5′- TGAGAAGGCGGATTCTGGATG - 3′) and ROP18-850-R (5′ - CCTTAACAGCCAACTCTTCATTCGTC - 3′). Primers specific to *T. gondii* actin (*TgACT1*) TgACT-F (5′ - TCCCGTCTATCGTCGGAAAG - 3′) and TgACT-R (5′ - CCATTCCGACCATGATACCC - 3′) were used as reference sample (internal control). Reaction mixtures containing 2 µL of cDNA and gene-specific primers were subjected to 40 thermal cycles (95°C for 15 sec and 60°C for 60 sec) of PCR amplification with the SmartCycler. Three replicate reactions were performed for each sample and values are reported as means. Threshold cycle (CT) values were calculated for *ROP18* using the SmartCycler software (Cepheid). Differences in the levels of gene expression for each of the strains were determined using the following formula. The amount of target gene (*ROP18*), normalized to the endogenous housekeeping gene (*TgACT1*), is given by 2^−ΔΔCT^, where ΔΔC_T_ = ΔC_T_ (sample)−ΔC_T_ (housekeeping), and ΔC_T_ is the C_T_ of the target gene subtracted from the C_T_ of the housekeeping gene, as described previously [51].

### Network Analysis of ROP18

A phylogenetic network of *ROP18* sequences was derived using the median-joining algorithm [52] (with ε = 0) as implemented in network 4.1 (www.flexus_engineeing.com). *ROP18* sequences from 37 strains of *T. gondii* (Table S2 plus additional strains listed in Table S4) were clustered using DNA Alignment v1.1.2.1 (www.flexus_engineeing.com). Aligned sequences were used to construct a network by combining the features of Kruskal's algorithm for finding minimum spanning trees that favors short connections and Farris's maximum-parsimony heuristic algorithm.

### Estimates of Most Recent Common Ancestry (MRCA)

The rate of mutation in *ROP18* was determined from a collection of 18 clonal isolates (i.e. types I, II and III) (Table S4). We only considered new mutations that arose since the common origin of the clonal types, hence biallelic polymorphisms that define the major allele types were excluded, as described previously [5]. This rate of diversity was compared to the occurrence of new polymorphisms in the antigen encoding genes *SAG1*, *SAG2* and a collection of introns used previously [5],[7]. Polymorphism rates were used to independently calculate the MRCA for the clonal lineages based on *ROP18*, antigens, and introns, respectively. For estimating the common ancestry based on coding sequences, we considered both 2-fold and 4-fold degenerate codons whereas for estimating the ancestry based on intron sequences, all nucleotide changes were calculated using the 4-fold rate (since these regions are noncoding all substitutions are 4-fold regenerate). This model makes the basic assumptions that each lineage is evolving independently and that mutations are accumulated randomly through time and that a Poisson distribution estimates the frequency of such rare events.

We estimated the MRCA for different alleles of *ROP18* using the synonymous polymorphisms observed between strains of different lineages given in Table S5. We grouped the strains into allele types in order to analyze the age of each *ROP18* allele type separately (i.e. I*, II*, and III*). Additionally we compared the alleles pairwise to estimate their divergence. Since we were interested in determining the common ancestry between alleles, we included all synonymous mutations including those that define biallelic polymorphisms between the alleles, as described previously [5]. The number of SNPs for the calculations was determined using equations presented in Table S5. Briefly, to determine ancestral *vs.* derived polymorphisms, we compared the *T. gondii* alleles to the coding region of *ROP18* from the *N. caninum* out-group. Alleles shared with *N. caninum* were considered to represent the ancestral state. Where *N. caninum* has diverged from *T. gondii* (no shared allele) we used a majority rule (i.e. the ancestral allele was considered to the be the allele shared by two strain types).

MRCA calculations were performed using the formula: t = S/(μ_a_Σn_i_l_i_+μ_b_Σn_i_m_i_), where n is the number of lineages examined at the i^th^ locus, l_i_ and m_i_ are the number of 4-fold and 2-fold synonymous sites, S is the number of polymorphisms and μ_a_ and μ_b_ are the neutral mutation rates. Two estimates of the neutral mutation rate from the closely related parasite *Plasmodium falciparum* were used for calculating the MRCA, as described previously [53],[54].

### Analysis of ROP18 in *Neospora caninum*

BLAST analysis identified a sequence 68% identical to the coding sequence of *T. gondii ROP18* in the *N. caninum* genome database (http://www.sanger.ac.uk/sequencing/Neospora/caninum/). The *ROP18* coding and upstream flanking sequences from the Nc-1 strain of *N. caninum* (ATCC# 50843) were confirmed by manual sequencing as described above. ClustalX/W was used to align the sequences using default settings. Phylogenetic comparisons were conducted on the coding regions of *ROP18* under the criterion of minimum evolution in PAUP*4.0b [55] and by using the BioNeighbor-Joining algorithm. A consensus tree was drawn according to bootstrap 50% majority rule with Nc-1 as the root.

### Comparison of ROP18 and Flanking Regions from *N. caninum* and *T. gondii* Strains

Genome sequence of *N. caninum* was obtained from The Wellcome Trust Sanger Institute (http://www.sanger.ac.uk/sequencing/Neospora/caninum/) by blasting the *T. gondii* ROP18 (ToxoDB gene id 20.m03896) and 2 flanking genes (20.m03897 and 20.m03895) (located on VIIa from1475116 to 1487487 bp) from ToxoDB (www.toxodb.org) [56]. This identified a ∼30 kb syntenic region from *N. caninum*. Pairwise comparisons of *T. gondii* and *N. caninum* sequences from this 40 kb region were carried out using AlignX (a component of Vector NTI Suite 9.0.0) (InforMax, Invitrogen Life Science Software, USA). Similarity values (in a 0–1 range) were assigned to each residue at a given alignment position in each aligned sequence, depending on whether the residue was identical, similar, weakly similar, or different.

## Supporting Information

Figure S1 Distribution of SNPs surrounding *ROP18*. Elevated levels of type III-specific single nucleotide polymorphisms (SNPs) occur within a 30 kb region of *ROP18*. A) Screen capture of the Genome browser view of *ROP18* (20.03896) and flanking regions from position 1449000 to 1489000 bp on chromosome VIIa (http://www.toxodb.org/). Annotated genes are shown in colored rectangles with corresponding gene ids. SNPs were identified by NUCmer alignments of GT1 (type I) and VEG (type III) to ME49 (type II) whole genome sequences and corresponding predicted coding regions. Red, green and blue color diamonds indicate the type I, II, and III specific SNPs, respectively. B) Graphical representation of type I (red), II (green), and III (blue) specific SNPs (in 1 kb windows) present on 40 kb region surrounding the ROP18 gene. Type III-specific SNPs are elevated throughout but show a strong peak for *ROP18*.
(0.39 MB PDF)

Table S1 List of loci used for diversity comparison of *T. gondii* strains.
(0.08 MB PDF)

Table S2 Strains used in this study.
(0.07 MB PDF)

Table S3 Comparison of pNS and pS ratios for *ROP18* alleles.
(0.07 MB PDF)

Table S4 Substitution rates and estimates of most recent common ancestor (MRCA) of the clonal lineages.
(0.08 MB PDF)

Table S5 Estimating the age of *ROP18* based on synonymous substitutions.
(0.08 MB PDF)
