# Supplementary figures and images for: Selection at a Single Locus Leads to Widespread Expansion of Toxoplasma gondii Lineages That Are Virulent in Mice
Source: PLoS Genet. 2009 Mar 6;5(3):e1000404. doi: 10.1371/journal.pgen.1000404 (PMC2644818; doi:10.1371/journal.pgen.1000404)

# A

## Chromosome VIIa

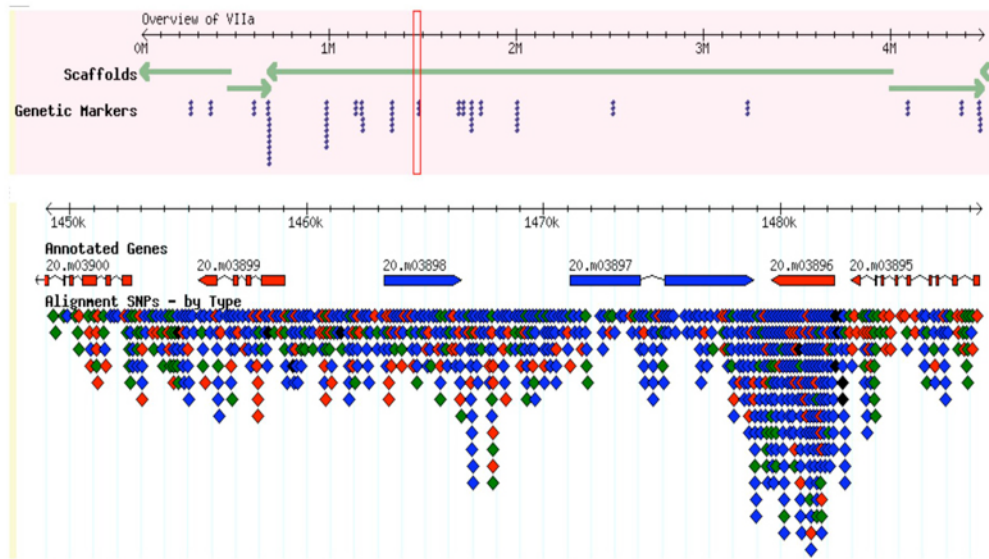

# B

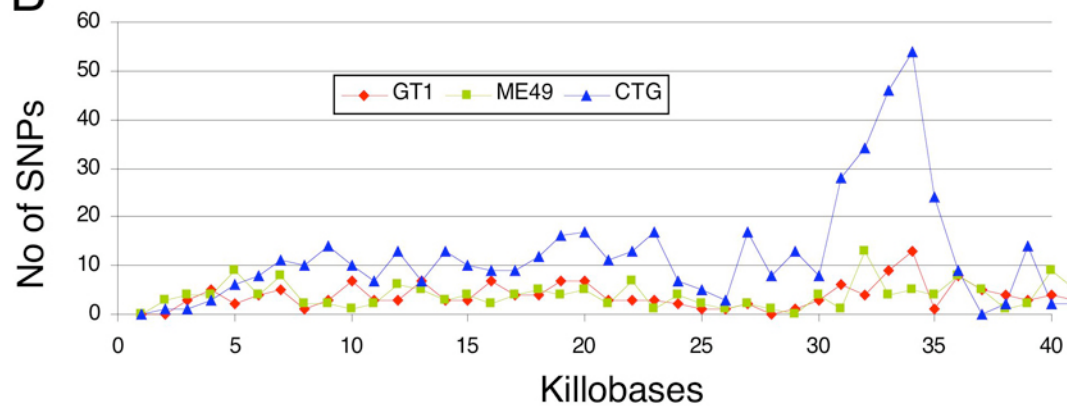

Supplement: Figure S1 — Distribution of SNPs surrounding ROP18. Elevated levels of type III-specific single nucleotide polymorphisms (SNPs) occur within a 30 kb region of ROP18. A) Screen capture of the Genome browser view of ROP18 (20.03896) and flanking regions from position 1449000 to 1489000 bp on chromosome VIIa (http://www.toxodb.org/). Annotated genes are shown in colored rectangles with corresponding gene ids. SNPs were identified by NUCmer alignments of GT1 (type I) and VEG (type III) to ME49 (type II) whole genome sequences and corresponding predicted coding regions. Red, green and blue color diamonds indicate the type I, II, and III specific SNPs, respectively. B) Graphical representation of type I (red), II (green), and III (blue) specific SNPs (in 1 kb windows) present on 40 kb region surrounding the ROP18 gene. Type III-specific SNPs are elevated throughout but show a strong peak for ROP18. (0.39 MB PDF) [file pgen.1000404.s001.pdf]
