# Supplementary material for: Selection at a Single Locus Leads to Widespread Expansion of Toxoplasma gondii Lineages That Are Virulent in Mice
Source: PLoS Genet. 2009 Mar 6;5(3):e1000404. doi: 10.1371/journal.pgen.1000404 (PMC2644818; doi:10.1371/journal.pgen.1000404)
Supplement: Table S1 — List of loci used for diversity comparison of T. gondii strains. (0.08 MB PDF) [file pgen.1000404.s002.pdf]

Table S1: List of loci used for diversity comparison of *T. gondii* strains

List of Loci	Annotation Gene Model[a]	Category	Chromosome
SAG1	44.m00009	Surface Antigens	VIII
SAG2	59.m00008	Surface Antigens	VIII
SAG3	551.m00001	Surface Antigens	XII
SAG4	72.m00004	Surface Antigens	VIIa
BSR4	641.m01561	Surface Antigens	IV
GRA2	42.m00015	Dense Granule	X
GRA3	42.m00013	Dense Granule	X
GRA4	583.m11414	Dense Granule	XI
GRA6	63.m00002	Dense Granule	X
GRA7	20.m00005	Dense Granule	VIIa
ROP1	583.m00003	Rhoptries	XI
ROP2	33.m01398	Rhoptries	X
ROP5	551.m00238	Rhoptries	XII
ROP7	83.m02145	Rhoptries	Ia
ROP8	33.m00005	Rhoptries	X
ROP16	55.m08219	Rhoptries	VIIb
ROP17	55.m08191	Rhoptries	VIIb
ROP18	20.m03896	Rhoptries	VIIa
BSR4 Related	86.m00375	Housekeeping	Ia
MIC2	20.m00002	Housekeeping	VIIa
AMA1	55.m00005	Housekeeping	VIIb
Actin (Actin)	25.m00007	Housekeeping	Ib
BTUB (β-tubulin)	57.m00003	Housekeeping	IX
ATUB	583.m00022	Housekeeping	XI
HSP90	49.m00060	Housekeeping	VI
BAG1/5	55.m00009	Housekeeping	VIIb
DHFR-TS	50.m00016	Housekeeping	XII
Hypothetical protein	42.m03409	Housekeeping	X
Hypothetical protein	42.m03493	Housekeeping	X
UPRT	583.m00018	Intron	XI
Elongation Factor intron 1	42.m00069	Intron	X
Hypothetical protein intron 2	27.m00099	Intron	IV

[a] Sequences from ToxoDB.org Gajria B, Bahl A, Brestelli J, Dommer J, Fischer S, et al. (2007) ToxoDB: an integrated *Toxoplasma gondii* database resource. Nucl Acids Res 36: D553-556.
