# Supplementary material for: Selection at a Single Locus Leads to Widespread Expansion of Toxoplasma gondii Lineages That Are Virulent in Mice
Source: PLoS Genet. 2009 Mar 6;5(3):e1000404. doi: 10.1371/journal.pgen.1000404 (PMC2644818; doi:10.1371/journal.pgen.1000404)
Supplement: Table S2 — Strains used in this study. (0.07 MB PDF) [file pgen.1000404.s003.pdf]

Table S2. Strains used in this study

Strain name	Host	Year	Geographic	Virulence[b]	Haplogroup[c]
RH	Human	1939	USA	100	1
GT1	goat	1980	USA-MD	100	1
ENT	Human (CT[a])	1985	France	100	1
VEL	Human (AIDS)	1988	USA	100	1
OH3	Human ocular	unknown	Brazil	100	1
MOR	Human (CT)	1988	France	100	1
Me49	Sheep	1965	USA-CA	40	2
DEG	Human (CT)	1987	France	0	2
PIH	Human (AIDS)	1992	USA	0	2
ARI	Human (Transplant)	1992	USA	80	2
PE	Human (Transplant)	1992	USA	31	2
B41	Bear	1994	USA	ND	2
CTG	Cat	1976	USA-NH	0	3
VEG	Human (AIDS)	1988	USA-CA	13	3
STRL	Human (CT)	unknown	USA	0	3
T61	Turkey	unknown	USA	ND	3
C56	Chicken	unknown	USA	0	3
M7741	Sheep	1958	USA	11	3
EDZ	Human (CT)	unknown	USA	ND	3
MAS	Human (CT)	1991	France (Nice)	100	4
TgCatBr1	Cat	2006	Brazil	90	4
TgCatBr18	Cat	2006	Brazil	100	4
CASTELLS	Sheep	1993	Uruguay	100	4
GUYKOE	Human	2002	French Guyana	100	5
GUYMAT	Human	2002	French Guyana	100	5
RUB	Human	1992	French Guyana	100	5
BOF	Human (AIDS)	1993	Belgium	8.3	6a
FOU	Human	1992	France (Brest)	100	6a
TgCatBr2	Cat	2006	Brazil	100	6b
TgCatBr9	Cat	2006	Brazil	100	6b
CAST	Human (AIDS)	1988	USA-CA	100	7
TgCatBr6	Cat	2006	Brazil	100	8
P89	Pig	1991	USA-IA	76	9
TgCatBr3	Cat	2006	Brazil	0	9
TgCatBr10	Cat	2006	Brazil	100	9
GUYDOS	Human	2001	French Guyana	100	10

VAND	Human	1997	French Guyana	100	10
COUG	Cougar	1996	Canada-BC	90	11

[a] Congenital

[b] % Cumulative mortality in outbred CD1 mice as defined previously)Taylor S, Barragan A, Su C, Fux B, Fentress SJ, et al. (2006) A secreted serine-threonine kinase determines virulence in the eukaryotic pathogen *Toxoplasma gondii*. Science 314: 1776-1780.)

[c] Khan A, Bohme U, Kelly KA, Adlem E, Brooks K, et al. (2006) Common inheritance of chromosome Ia associated with clonal expansion of *Toxoplasma gondii*. Gen Res 16: 1119-1125.

Khan A, Fux B, Su C, Dubey JP, Darde ML, et al. (2007) Recent transcontinental sweep of *Toxoplasma gondii* driven by a single monomorphic chromosome. Proc Natl Acad Sci (USA) 104: 14872-14877.

ND, not determined
