# Supplementary material for: Selection at a Single Locus Leads to Widespread Expansion of Toxoplasma gondii Lineages That Are Virulent in Mice
Source: PLoS Genet. 2009 Mar 6;5(3):e1000404. doi: 10.1371/journal.pgen.1000404 (PMC2644818; doi:10.1371/journal.pgen.1000404)
Supplement: Table S3 — Comparison of pNS and pS ratios for ROP18 alleles. (0.07 MB PDF) [file pgen.1000404.s004.pdf]

Table S3 Comparison of pNS and pS ratios for *ROP18* alleles

Strains	*ROP18*		
	pNS	pS	pNS/pS
[a]Type I vs. Type II	0.02497	0.00536	4.660258
Type I vs. Type III	0.06049	0.03473	1.742024
Type II vs. Type III	0.05415	0.04036	1.341758
[b]Type I* vs. Type II*	0.02323	0.00675	3.535183
Type I* vs. Type III*	0.06741	0.04565	1.476658
Type II* vs. Type III*	0.05665	0.04895	1.159953
[c]Type I*a vs. Type I*b	0.00238	0.00252	0.944776
Type I*a vs. Type I*c	0.00383	0.00251	1.527424
Type I*a vs. Type I*c	0.00621	0.00503	1.236186

pN, Estimate of the proportion of nonsynonymous differences; pNS = NSd/NS,
NS, The number of nonsynonymous sites for each codon
NSd, The number of synonymous differences per codon
pS, Estimate of the proportion of synonymous differences; pS = Sd/S,
S, The number of synonymous sites for each codon
Sd, The number of synonymous differences per codon
[a] Includes only North American and European clonal lineages, based on 2 strains each.
[b] ROP18I* (17) II* (3) and III* (5) groups were defined based on the phylogeny shown in Fig 2C and the numbers of strains are given in parentheses.
[c] ROP18Ia* (5), ROP18I*b (5) and ROP18I*c (6) are defined in Figure 4A and the numbers of strains are given in parentheses.
