# Supplementary material for: Selection at a Single Locus Leads to Widespread Expansion of Toxoplasma gondii Lineages That Are Virulent in Mice
Source: PLoS Genet. 2009 Mar 6;5(3):e1000404. doi: 10.1371/journal.pgen.1000404 (PMC2644818; doi:10.1371/journal.pgen.1000404)
Supplement: Table S4 — Substitution rates and estimates of most recent common ancestor (MRCA) of the clonal lineages. (0.08 MB PDF) [file pgen.1000404.s005.pdf]

Table S4 Substitution rates and estimates of most recent common ancestor (MRCA) of the clonal lineages

A. Protein coding genes

Gene	Groups	Total	Sequenced Region			Synonymous polymorphisms	Age in years since TMRCA		No of Strains	Haplo-group
			Two fold degenerate sites	Four fold degenerate sites	Total bp		M1	M2		
ROP18	Type I	Total adj	1374	1710	10026	0	NA	NA	6	1
		Total obs	1374	1710	10026	2.99[¶]	6.56 X 10^5	3.28 X 10^5	6	1
	Type II	Total adj	1143	1427	8355	0	NA	NA	5	2
		Total obs	1143	1427	8355	2.99[¶]	7.87 X 10^5	3.93 X 10^5	5	2
	Type III	Total adj	1362	1740	10026	0	NA	NA	6	3
		Total obs	1362	1740	10026	2.99[¶]	6.51 X 10^5	3.24 X 10^5	6	3
SAG1	Type I	Total adj	726	1134	6066	3	1.07 X 10^6	5.27 X 10^5	6	1
		Total obs	726	1134	6066	7.75[¶¶¶]	2.77 X 10^6	1.36 X 10^6	6	1
	Type II	Total adj	610	945	5055	0	NA	NA	5	2
		Total obs	610	945	5055	2.99[¶]	1.28 X 10^6	6.29 X 10^5	5	2
	Type III	Total adj	732	1134	6066	0	NA	NA	6	3
		Total obs	732	1134	6066	2.99[¶]	1.06 X 10^6	5.25 X 10^5	6	3
	Type I	Total adj	384	702	3384	0	NA	NA	6	1
		Total obs	384	702	3384	2.99[¶]	1.80 X 10^6	8.80 X 10^5	6	1

SAG2	Type II	Total adj	325	585	2820	0	NA	NA	5	2
		Total obs	325	585	2820	2.99[¶]	2.16 X 10^{6}	1.05 X 10^{6}	5	2
	Type III	Total adj	384	702	3384	0	NA	NA	6	3
		Total obs	384	702	3384	2.99[¶]	1.81 X 10^{6}	8.80 X 10^{5}	6	3

B. Introns

Introns	Groups	Total bp[a]	Polymorphisms		M1*	M2*	No of Strains	Haplogrops
			SNPs	Indels	1.7 X 10^{-9}	3.8 X 10^{-9}		
					SNPs	SNPs		
UPRT, BTUB, MIC2, HP and EF1	Type I	15140	1	1	3.8 X 10^{4}	1.7 X 10^{4}	4	1
			4.74[¶¶]	4.74[¶¶]	1.8 X 10^{5}	8.2 X 10^{4}	4	1
	Type II	11355	0	0	NA	NA	3	2
			2.99[¶]	2.99[¶]	1.5 X 10^{5}	6.9 X 10^{4}	3	2
	Type III	11355	1	0	5.2 X 10^{4}	2.3 X 10^{4}	3	3
			4.74[¶¶]	2.99[¶]	2.4 X 10^{5}	1.1 X 10^{5}	3	3

NA, Not applicable, M1 (Hughes AL, Verra F (2001) A very large long-term effective population size in the virulent human malaria parasite *Plasmodium falciparum*. Proc Roy Soc Lond 268: 1855-1860.), and M2 (Rich SM, Licht MC, Hudson RR, Ayala FJ (1998) Malaria's Eve: evidence for a recent population bottleneck throughout the world populations of *Plasmodium falciparum*. Proc Natl Acad Sci (USA) 95: 4425-4430.), are different age estimates for the neutral mutation rate

[¶] Total adjusted (Total adj) was calculated by using Poisson upper 95% confidence limit for zero observation: 2.99 “total observed” (Total obs) mutations

[¶¶] Total adjusted (Total Adj) was calculated by using Poisson upper 95% confidence limit for 2 observations: 6.29 "total observed" (total obs) mutations

[¶¶¶] Total adjusted (Total Adj) was calculated by using Poisson upper 95% confidence limit for 3 observations: 7.75 "total observed" (total obs) mutations

[a] All sites calculated as four fold degenerate since they are noncoding.

M1*, Mutation rate at four fold degenerate sites., M2*, Mutation rate at four fold degenerates sites.

MRCA Calculation: Total synonymous number of SNPs / (Two fold codon sites x Number of strains x Two fold mutation rate) + (Four fold codon sites x Number of strains x Four fold mutation rate)

Strains used:

Type I: RH, GT1, ENT, VEL, OH3, MOR

Type II: ME49, DEG, PIH, ARI, B41

Type III: CTG, VEG, STRL, T61, EDZ, M7741
