# Supplementary material for: Selection at a Single Locus Leads to Widespread Expansion of Toxoplasma gondii Lineages That Are Virulent in Mice
Source: PLoS Genet. 2009 Mar 6;5(3):e1000404. doi: 10.1371/journal.pgen.1000404 (PMC2644818; doi:10.1371/journal.pgen.1000404)
Supplement: Table S5 — Estimating the age of ROP18 based on synonymous substitutions. (0.08 MB PDF) [file pgen.1000404.s006.pdf]

Table S5 Estimating the age of *ROP18* based on synonymous substitutions

**Groups**	**Total SNPs**	**Sequenced Region**			**Synonymous Polymorph-isms**	**Age in years since TMRCA**		**No of Strains**	**Haplo-groups**
		Two fold degenerate sites	Four fold degenerate sites	Total bp		M1	M2		
[a]Type I*	Total obs	4799	5986	35091	11	6.90×10^5	3.45×10^5	21	1, 4, 5, 6, 7, 8, 9, 10
Type II*	Total obs	1371	1713	10026	0	NA	NA	6	2, 11
	Total adj	1371	1713	10026	2.99[¶]	6.56×10^5	3.28×10^5		
Type III*	Total obs	2270	2899	15039	2	2.61×10^5	1.30×10^5	9	3, 9, 4
	Total adj	2270	2899	15039	6.29[¶¶]	8.22×10^5	4.10×10^5		
Type I* vs Type II*[b]	Total obs	6170	7699	45117	23	1.12×10^6	5.61×10^5	27	1, 4, 5, 6, 7, 8, 9, 10, 2, 11
Type I* vs Type III*[b]	Total obs	7069	8885	50130	283	1.2×10^7	6×10^6	30	1, 4, 5, 6, 7, 8, 9, 10, 3, 9, 4
Type II* vs Type III*[b]	Total obs	3641	4612	26736	290	2.37×10^7	1.19×10^7	16	2, 11, 3, 9, 4

[a] As defined in Fig. 2C, NA, Not applicable, M1 (Hughes AL, Verra F (2001) A very large long-term effective population size in the virulent human malaria parasite *Plasmodium falciparum*. Proc Roy Soc Lond 268: 1855-1860.), and M2 (Rich SM, Licht MC, Hudson RR, Ayala FJ (1998) Malaria's Eve: evidence for a recent population bottleneck throughout the world populations of *Plasmodium falciparum*. Proc Natl Acad Sci (USA) 95: 4425-4430.), are different age estimates for the neutral mutation rate.

[¶] Total adjusted (Total adj) was calculated by using Poisson upper 95% confidence limit for zero observation: 2.99 "total observed" (Total obs) mutations, limit for 2 observations: 6.29 "total observed" (total obs) substitutions.

MRCA Calculation: Total synonymous number of SNPs / (Two fold codon sites x Number of strains x Two fold mutation rate) + (Four fold codon sites x Number of strains x Four fold mutation rate)

[b]Rules used for classifying SNPs: Type III = Neospora = Ancestral state, Type I/II = Neospora = Ancestral state, Type III not equal to Neospora, the majority is the Ancestral state.
